# Disposing of Unwanted Firearms and Firearm Injury Prevention

**DOI:** 10.1001/jamanetworkopen.2024.41606

**Published:** 2024-10-28

**Authors:** David K. Humphreys, Douglas J. Wiebe

**Affiliations:** 1 Department of Social Policy and Intervention, University of Oxford, Oxford, Oxfordshire, United Kingdom; 2 Department of Emergency Medicine, University of Michigan, Ann Arbor

## Abstract

This comparative effectiveness study estimates the size of the US firearm stock by 2034.

## Introduction

The US firearm stock is estimated to be over 393 million firearms.^1^ Strategies for mitigating harms associated with high firearm availability include access and usage restrictions and safe storage practices.^2^ However, even if enacted, these initiatives would have to contend with an ever-increasing number of firearms in circulation. Every year, millions of new firearms are produced and purchased—firearms that remain functional for decades and likely outlast their primary owners. This study forecasts the growth of the US civilian firearm stock by 2034, estimating how minor changes in firearms leaving circulation (ie, the attrition rate) could change its future size.

## Methods

In this comparative effectiveness research study, we used time-series forecasting to estimate the size of the US firearm stock by 2034. We obtained annual counts of firearms manufactured for the US market between 1946 and 2022 from the Bureau of Alcohol, Firearms, Tobacco, and Explosives (ATF). We estimated the number of firearms currently in circulation using the total number of firearms manufactured minus exports and a 1% annual attrition rate to account for the number of firearms leaving the firearm stock each year (eg, destruction, damage, decay, and so forth).^3^ To forecast the firearm stock between 2022 to 2034, we applied autoregressive integrated moving-average (ARIMA) time-series models to historical data, then applied hypothetical scenarios to estimate the size of the 2034 civilian firearm stock under different attrition rate scenarios (1.5%, 2.0%, 2.5%, and 3.0%) (see eMethods in Supplement 1). Ethical approval and the need for informed consent were waived by the institutional review board of University of Michigan and determined to be exempt because the research did not involve human participants. We followed the International Society for Pharmacoeconomics and Outcomes Research (ISPOR) reporting guidelines. The Ljung-Box test was used to assess autocorrelation in the residuals, *P* values were 1-sided, and a significance level of .05 was applied. Data were analyzed in R version 4.3.0 (R Project for Statistical Computing) from April to May 2024.

## Results

We estimate that the cumulative number of firearms in circulation has increased from 45 million in 1946 to 378 million in 2022 (Figure, A). This analysis estimates that, at its present trajectory, the firearm stock could reach 565 million firearms by 2034 (95% prediction interval [PI], 506.0-623.3 million)—a 50% increase on the 2022 firearm stock (Figure, B). This analysis suggests that modest increases in the annual attrition rate, for example from 1% to 3%, could result in over 115 million fewer firearms in circulation by 2034 (Table and Figure, C).

**Figure.**
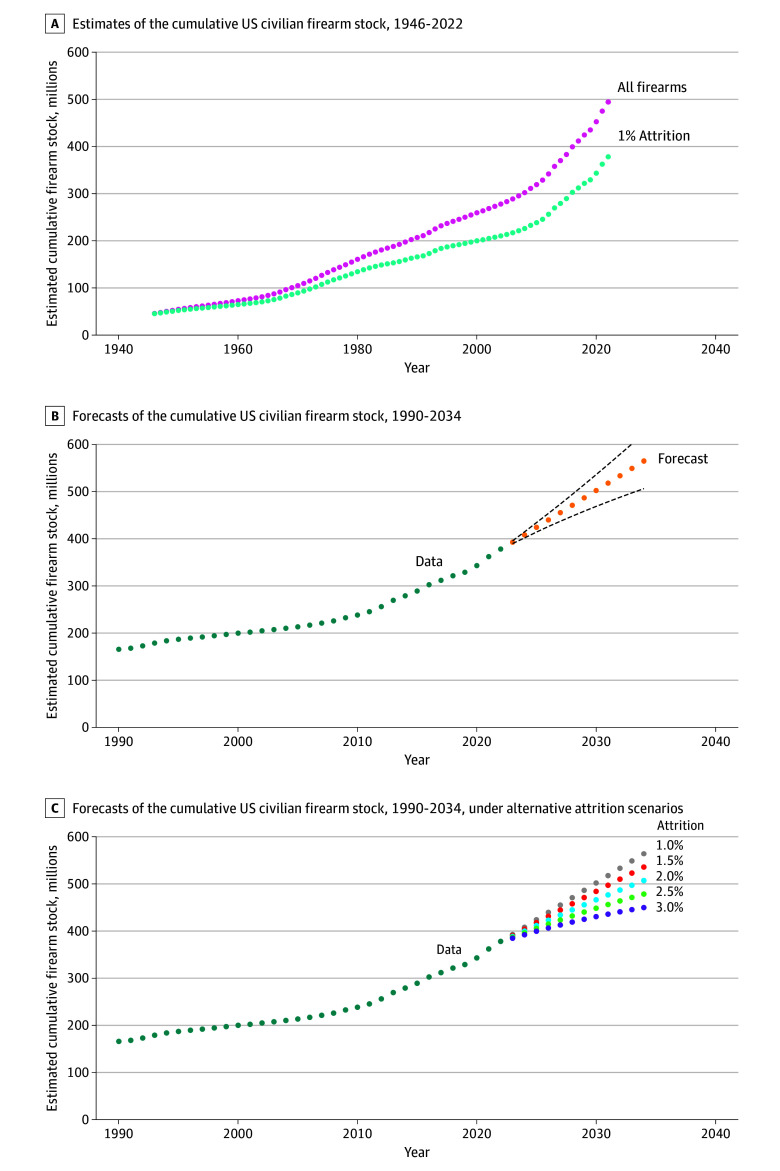
Estimating Future Civilian Firearm Stock A, Estimated firearm stock from ATF firearm manufacturing figures. The pink line represents the number of firearms manufactured for the US market, excluding exports. The blue line adjusts these figures for a 1.0% attrition rate. B, Forecasted trajectory of the US civilian firearm stock, based on historical trends and patterns in the data, and accounting for a 1.0% attrition. Green data points depict actual records of firearms entering the US market, while orange data points show the projected values. The dashed lines show the 95% prediction intervals. C, Hypothetical projections for future size of the civilian firearm stock under various assumptions of changes to the attrition rate after 2022. Gray points show projections where attrition remains at 1.0%, red points at 1.5%, blue at 2.0%, green at 2.5%, and purple at 3.0%.

**Table.** Results of Autoregressive Integrated Moving-Average (ARIMA) Forecasts^a^

Post-2022 annual attrition scenario	No. (millions) of firearms estimated in circulation by 2034 (95% prediction interval)	Estimated change from 1% attrition	
		No.	% Change
1.0% (Baseline assumption)	564.7 (506.0-623.3)	NA	NA
1.5%	536.0 (479.0-592.9)	−28.7	−5.1
2.0%	507.2 (451.9-562.5)	−57.4	−10.2
2.5%	478.5 (424.9-532.1)	−86.2	−15.3
3.0%	449.8 (397.8-501.7)	−114.9	−20.3

Abbreviations: AIC, Akaike information criterion; AR, autoregressive component; NA, not applicable; Q, Ljung-Box Q statistic.

^a^ ARIMA (p,d,q), 2,2,0; AR1 (SE), −0.02 (0.11); AR2 (SE), −0.32 (0.12); AIC, 276.26; Ljung-Box Q, 9.14; *P* = .33. In the ARIMA model, p indicates the autoregressive order, or the number of lag observations included in the model; d indicates the differencing order, or the number of times the data has been differenced to make it stationary; q indicates moving average order, or the number of lagged forecast errors in the model.

## Discussion

There are well-known challenges when estimating firearm ownership in the US. We used ATF data to generate historical estimates of the cumulative firearm stock. These data are imperfect but provide reliable estimates compared with findings from nationally representative surveys.^4^ Our findings project a dramatic increase in the size of the firearm stock over the next decade, which could pose a considerable increase in risk to public safety. However, modest increases to the rate of firearm attrition could moderate the size of the 2034 firearm stock, and thus reduce risks to public safety.

Increasing attrition rates may be possible through enhancing efforts to help individuals voluntarily dispose of unwanted firearms. At present, services, infrastructure, and information to support individuals wishing to dispose of unwanted firearms are severely lacking. Local initiatives (eg, buyback programs) are costly and implemented unpredictably across jurisdictions, forcing many individuals to retain firearms they do not want.^5^ Little is known about the proportion of the firearm stock that may be unwanted, or what motivates individuals to divest from their firearms. Nationally representative surveys suggest that over a quarter of firearm owners acquire firearms through nonpurchase transfers (ie, inheritance, gifts, and so forth), meaning that large proportions of owners acquire firearms passively, rather than intentionally.^4^ With over a third of the civilian firearm stock concentrated in older age groups (≥60 years),^6^ there are opportunities to address the number of firearms in circulation by improving services to support those inheriting or otherwise receiving firearms they do not want.

## Notes

Supplement 1.
eMethods.

eReferences.

Supplement 2.
Data Sharing Supplement
