# Supplementary material for: Disposing of Unwanted Firearms and Firearm Injury Prevention
Source: JAMA Netw Open. 2024 Oct 28;7(10):e2441606. doi: 10.1001/jamanetworkopen.2024.41606 (PMC11519752; doi:10.1001/jamanetworkopen.2024.41606)
Supplement: Supplement 1. — eMethods. eReferences. [file jamanetwopen-e2441606-s001.pdf]

Supplemental Online Content

Humphreys DK, Wiebe DJ. Disposing of unwanted firearms and firearm injury prevention. *JAMA Netw Open*. 2024;7(10):e2441606. doi:10.1001/jamanetworkopen.2024.41606

eMethods

eReferences

This supplemental material has been provided by the authors to give readers additional information about their work.

© 2024 Humphreys DK et al. *JAMA Network Open*.

eMethods

1. Rationale

Recent polls find that sixty percent of Americans consider societal firearm violence to be a 'big problem', with only four percent indicating it is 'not a problem at all'.[1] Present policy options consider a limited range of initiatives acceptable within the narrow confines of political debates, including some access restrictions, safe storage practices, and firearm usage policies.[4,5] But even if these interventions could be widely enacted, it would be against a backdrop of an increasing civilian firearm stock. Many millions of new firearms are purchased each year and will remain functional for decades due to their durability.[6,7] In this analysis we apply time-series forecasting methods to predict the growth of the civilian firearm stock over the next ten years. We examine different scenarios of firearm attrition (i.e. the annual removal of firearms from the civilian stock) to estimate how hypothetical changes in the rates of firearms leaving the system might impact the overall size of the firearm stock by 2034.

2. Methods

2.1 Objective

To predict the size of the US civilian firearm stock by the year 2034 and consider options to mitigate harm from increases in the availability of firearms.

2.2 Study Design

We use a predictive time-series design to forecast the size of the firearm stock by 2034. We use the estimates from these models to project the trajectory and size of the civilian firearm stock by 2034 under several different hypothetical scenarios in which attrition rates (i.e. removal of firearms from society) are modified. We followed ISPOR reporting guidelines for reporting the design and methodology and discussing the limitations of the study.

2.3 Data

One of the few resources available to approximate numbers of firearms in circulation is the Annual Firearms Manufacturing and Exportation Report[1], published by the Bureau of Alcohol, Firearms and Tobacco (ATF). These data capture the number of firearms manufactured for the US market from 1899 to present. Figures are presented as annual manufactured numbers of

---

[1] An online spreadsheet of these data were made available by the news organization *The Trace*: https://docs.google.com/spreadsheets/d/e/2PACX-1vShiwPP36HrjUyPztuIhoYjR3Xd3uhlQivMC3wPSNq1UCjVbj2wsPZIISqOtFQ0NbVVwwKBzyrDNuCz/pubhtml#

© 2024 Humphreys DK et al. *JAMA Network Open*.

firearms, together with the number of firearms exported and imported, disaggregated by firearm type (i.e. shotguns, rifles, long guns, miscellaneous). Estimates of the number of firearms in circulation are calculated by adding the total number firearms manufactured or imported and subtracting firearms exported each year. Because production figures potentially overestimate the total number of firearms in circulation experts have applied a one percent annual attrition, as standard, to adjust for the number of firearms leaving the firearm stock (e.g. due to damage, decay, confiscation and destruction etc).[8,9] This attrition rate is applied to figures of firearms produced and imported to the US market to enable estimation of the number of firearms in circulation. In the absence of more sophisticated licensing and registration data—that exist in some other countries[12]—estimates derived from ATF data have been shown to have reasonable face validity when compared with estimates of the civilian firearm stock derived from population representative surveys.[8,9]

2.4 Main Outcomes and Measures

Cumulative annual numbers of firearms (adjusted for attrition) estimated to be in circulation for the US market between 1946 and 2022.

2.5 Exposure

This study uses a hypothetical exposure to represent different attrition rates (i.e. annual rate of firearms leaving the civilian stock). Using the standard 1 percent attrition level applied in other studies, this study asks what the size of the civilian firearm stock might look like under different annual attrition rates from 2023 onwards. This thought experiment simulates how modest efforts to increase the rate at which firearms leave the civilian firearm stock might moderate its size over the next decade. In this paper we follow previous studies by using a one percent attrition rate to estimate the overall size and growth of the current civilian firearm stock.[9] Using this as a basis for our counterfactual project of future size of the firearm stock, we created four hypothetical scenarios in which rates of firearm attrition could increase from 2022 onwards. In an effort to consider permutations within a realistic band of possible change we considered four modest increases in attrition: 1.5%, 2%, 2.5% and 3%.[11]

2.6 Analysis

Autoregressive integrated moving-average (ARIMA) time-series models were developed to forecast the growth of the civilian firearm stock between 2022 to 2034. ARIMA models can be used to provide *n*-step ahead predictions, that are based on temporal dependence in time

© 2024 Humphreys DK et al. *JAMA Network Open*.

series data. ARIMA models are composed of three components (*p, d, q*) representing the key features of time-series data, namely *autocorrelation* (*p*), differencing required for *stationarity* (d), and *moving-average* (q). We used the Box-Jenkins methodology for model estimation and diagnosis.[13] Autocorrelation and partial autocorrelation plots were used to examine autocorrelation and Box-Lung tests examined stationarity and white noise in residuals. To optimise forecasts, we created a training dataset of 70 data points (years from 1946 to 2015), with the remaining seven (approximately ten percent) of data points used to cross-validate forecast accuracy. Forecasts were evaluated using visual methods (e.g. one-to-one plots) and through statistical tests including root-mean squared error (RMSE). Time-series forecasts can be susceptible to bias due to exogenous shocks. There are numerous reasons why manufacturing of firearms may be sensitive to external factors and there is growing evidence that demand (and production) shift in response to societal events.[14,15] We used statistical detection methods to identify structural breaks in times series data. For this we used the 'strucchange' package in R v.3.[16,17] Structural breaks tests detected gradient changes in the outcome variable for six different periods: 1946-1966, 1967-1977, 1978-1988, 1989-1999, 2000-2010, and 2011-2022. Using these detected breaks in the historical trends, we created dummy variables for each event and incorporated them as independent variables in adjusted forecasts. Results for adjusted forecasts were comparable to the unadjusted models. The results for the adjusted models can be provided upon request. All analyses were conducted in R v 4.3.0.

© 2024 Humphreys DK et al. *JAMA Network Open*.

eReferences

1. Schaeffer K. Key facts about Americans and guns [Internet]. Pew Research Center. 2023 [cited 2024 Apr 24]. Available from: https://www.pewresearch.org/short-reads/2023/09/13/key-facts-about-americans-and-guns/

2. Boine C, Siegel M, Ross C, Fleegler EW, Alcorn T. What is gun culture? Cultural variations and trends across the United States. Humanit Soc Sci Commun. 2020 Jul 8;7(1):1–12.

3. Yamane D. The sociology of U.S. gun culture. Sociol Compass. 2017;11(7):e12497.

4. Roche JS, Carter PM, Zeoli AM, Cunningham RM, Zimmerman MA. Challenges, Successes, and the Future of Firearm Injury Prevention. Milbank Q. 2023;101(S1):579–612.

5. Swanson JW. Preventing Suicide Through Better Firearm Safety Policy in the United States. Psychiatr Serv. 2021 Feb;72(2):174–9.

6. Steidley T, Kosla MT. Toward a Status Anxiety Theory of Macro-level Firearm Demand. Soc Curr. 2018 Feb 1;5(1):86–103.

7. Sorenson SB. Regulating Firearms as a Consumer Product. Science. 1999 Nov 19;286(5444):1481–2.

8. Azrael D, Hepburn L, Hemenway D, Miller M. The Stock and Flow of U.S. Firearms: Results from the 2015 National Firearms Survey. RSF. 2017 Oct 1;3(5):38–57.

9. Cook PJ, Ludwig J. Guns in America: National Survey on Private Ownership and Use of Firearms. Washington D.C.: U.S. Departmen of Justice; 1997. (National Institute of Justice: Research in Brief).

10. Legault RL, Hendrix N, Lizotte AJ. Caught in a crossfire: legal and illegal gun ownership in America. In: Handbook on Crime and Deviance. Switzerland: Springer Nature; 2009. (Handbooks of Sociology and Social Research).

11. Mascia J, Brownlee C. How Many Guns Are Circulating in the U.S.? [Internet]. The Trace. 2023 [cited 2024 Feb 6]. Available from: https://www.thetrace.org/2023/03/guns-america-data-atf-total/

12. Karp A. Estimating Global Civilian-Held Firearms Numbers. Geneva, Switzerland: Small Arms Survey; 2018.

13. Box GEP, Jenkins GM, Reinsel GC, Ljung GM. Time Series Analysis: Forecasting and Control. John Wiley & Sons; 2015. 709 p.

14. Depetris-Chauvin E. Fear of Obama: An empirical study of the demand for guns and the U.S. 2008 presidential election. J Public Econ. 2015 Oct 1;130:66–79.

15. Schleimer JP, McCort CD, Shev AB, Pear VA, Tomsich E, De Biasi A, et al. Firearm purchasing and firearm violence during the coronavirus pandemic in the United States: a cross-sectional study. Inj Epidemiol. 2021 Jul 5;8(1):43.

16. Zeileis A, Kleiber C, Krämer W, Hornik K. Testing and dating of structural changes in practice. Comput Stat Data Anal. 2003 Oct 28;44(1):109–23.

© 2024 Humphreys DK et al. *JAMA Network Open*.

17. Zeileis A, Leisch F, Hornik K, Kleiber C. strucchange: An R Package for Testing for Structural Change in Linear Regression Models. J Stat Softw. 2002 Jan 10;7:1–38.

© 2024 Humphreys DK et al. *JAMA Network Open*.
