# Supplementary material for: Disposing of Unwanted Firearms and Firearm Injury Prevention
Source: JAMA Netw Open. 2024 Oct 28;7(10):e2441606. doi: 10.1001/jamanetworkopen.2024.41606 (PMC11519752; doi:10.1001/jamanetworkopen.2024.41606)
Supplement: Supplement 2. — Data Sharing Supplement [file jamanetwopen-e2441606-s002.pdf]

Data Sharing Statement

Humphreys. Disposing of Unwanted Firearms and Firearm Injury Prevention. *JAMA Netw Open*. Published October 28, 2024. doi:10.1001/jamanetworkopen.2024.41606

Data
Data available: Yes
Data types: Other (please specify)
Additional Information: Data is in the public domain.
How to access data: Data and analytical code will be uploaded to GitHub upon acceptance.
When available: With publication

Supporting Documents
Document types: Statistical/analytic code
How to access documents: Code will be made available to GitHub. Requests can be made to: David.humphreys@spi.ox.ac.uk
When available: With publication

Additional Information
Who can access the data: Anyone requesting data
Types of analyses: Any purposes
Mechanisms of data availability: With investigator support.
